# UNDERUTILIZATION OF HOSPICE CARE IN OLDER BLACK ADULTS

**DOI:** 10.1093/geroni/igad104.2371

**Published:** 2023-12-21

**Authors:** Channing Tate

**Affiliations:** University of Colorado Anschutz Medical Campus, Aurora, Colorado, United States

## Abstract

Hospice is underutilized in Black Americans despite evidence that Black Americans benefit from hospice services. There are several potential reasons hospice is underutilized in Black Americans including lack of knowledge of hospice, poor opinions of hospice, and low self-efficacy in end-of-life decision-making. Patient decision aids are one technique used to improve patient knowledge and agency in making medical decisions. This research project endeavored to addresses potential barriers to hospice enrollment in Black adults and evaluates if a hospice specific patient decision aid could improve hospice knowledge, opinions of hospice, and decision-making self-efficacy. The study was a pilot randomized controlled trial that enrolled Black adults aged ≥ 65 years. The three primary outcomes for the study included hospice knowledge measured by the Hospice Knowledge Scale, opinions of hospice measured by the Hospice Beliefs and Attitudes Scale, and confidence in making decisions measured by the Decision Self-Efficacy Scale. Additionally acceptability and usability outcomes of the decision aid were collected. All participants completed the three baseline surveys prior to randomization. Those randomized to the intervention were provided with the hospice patient decision aid while those in the control did not receive the decision aid. Hospice knowledge, opinions of hospice, and decision self-efficacy improved from baseline to one-month follow-up in the intervention group, but between group (intervention vs control) differences were not statistically significant. Overall acceptability outcomes were favorable, and participants stated the patient decision aid would be beneficial in facilitating hospice decision making.

